# An outbreak of RSV infections in a neonatology clinic during the RSV-season

**DOI:** 10.1186/s12887-021-03053-9

**Published:** 2021-12-11

**Authors:** Liliya Vakrilova, Stanislava Hitrova Nikolova, Sergei Slavov, Petya Radulova, Boryana Slancheva

**Affiliations:** 1 Department of Obstetrics and Gynecology, Medical University of Sofia, Zdrave 2 street, 1431 Sofia, Bulgaria; 2 Neonatology Clinic, University Hospital of Obstetrics and Gynecology “Maichin Dom”, Sofia, Bulgaria; 3 Gynecology Department, University Hospital of Obstetrics and Gynecology “Maichin Dom”, Sofia, Bulgaria

**Keywords:** RSV-infections, Preterm infants, Term infants, NICU, Palivizumab

## Abstract

**Background:**

Respiratory syncytial virus (RSV) is the predominant cause of lower respiratory tract infections (LRTI) in infancy. Preterm infants with bronchopulmonary dysplasia (BPD) are at the highest risk of severe RSV-LRTI. This is a retrospective study that analyses a nosocomial outbreak of RSV infections in the Neonatology clinic of the University Hospital of Obstetrics and Gynecology, Sofia, 2019.

**Methods:**

Two groups of infants without contact between them were diagnosed with RSV-infection: 14 infants were treated in the Department for healthy newborns – Group 1, and 7 preterm infants were treated in the Neonatal Intensive Care Unit (NICU) – Group 2. The detection of RSV was performed using Real-Time PCR in nasal/throat swabs.

**Results:**

Respiratory symptoms occurred 2–5 days after discharge in 14 of 148 healthy term infants born February 5 to 18, 2019; 12 babies were re-hospitalized with LRTI and recovered in a few days. RSV-PCR was positive in 6 infants, while in the others, RSV etiology was suggested, due to similar symptoms and contact between them.

The first NICU patient with RSV-LRTI was one of the 26 gestational weeks (GW) twins, who had severe BPD. The other twin was always discharged home without LRTI-symptoms. In the period February 19 to March 15, 2019, 26 premature babies born at 26–34 GW, were tested for RSV (33 nasal/throat swabs). They received a first or subsequent palivizumab injection. We identified 11 positive samples in 7 of the babies. Despite the clinical recovery, the second RSV-PCR remained positive in 4 babies. Six of the 7 NICU patients had symptoms of LRTI, and two of them needed mechanical ventilation. Six babies were discharged home after stabilization, one was transferred to the Pediatric department for further treatment of BPD and later discharged too.

**Conclusions:**

This was the most serious outbreak of RSV infections in neonates since the RSV-PCR diagnostic in Bulgaria was introduced. The course of RSV-LRTI was severe in extremely preterm patients with underlying BPD. So, routine in-hospital RSV-prophylaxis with palivizumab should be considered for infants at the highest risk.

## Background

Respiratory syncytial virus (RSV) is the most common etiologic agent for acute respiratory infections overall and for lower respiratory tract infections (LRTI) - bronchiolitis and pneumonia in infants. RSV causes between 50,000 and 125,000 annual hospitalizations of US children younger than 5 years [1, 2]. In 2015, RSV was responsible for 33.1 million LRT infections worldwide, approximately 3.2 million hospital admissions, and 59,600 in-hospital deaths in children younger than 5 years [3]. RSV is a leading cause of hospitalization for infants less than 2 years of age [4–6]. Risk factors for severe illness and mortality are preterm birth, chronic lung disease (CLD), hemodynamically significant congenital heart disease (CHD), age less than 3 months, neuromuscular disorders, and immunodeficiency [4, 7]. Healthy full-term or near-term infants of 34–37 gestational weeks (GW), can be affected too [8]. RSV is mainly transmitted by aerosols or direct contact with contaminated surfaces where the virus can remain virulent for hours. The incubation period is between 4 and 5 days with initial viral replication in the nasopharynx; thereafter the virus can cause LRTI [9]. As a highly contagious pathogen, RSV carries a high risk of nosocomial spreads in Neonatal Intensive Care Units (NICU) and RSV outbreaks are more frequent than suspected [10].

There is currently no licensed vaccine to prevent RSV infection, but in the past two decades, passive immunoprophylaxis for high-risk infants was increasingly implemented using a monoclonal antibody, palivizumab [11, 12]. This reduced the risk of RSV-LRTI hospitalizations for these infants [13, 14].

In Bulgaria, RSV was the most frequently detected etiologic agent of LRTI in children under the age of 5 for the seasons 2014/15, 2015/16, 2016/17, 2017/18 [15, 16]. The palivizumab prophylaxis started in 2010 for the most vulnerable groups with a gradual extension of the indications in the subsequent years. To date, there are no national guidelines for the use of palivizumab in NICUs, or control of in-hospital RSV outbreaks.

## Methods

This study reports retrospectively data on case series, and analyses the nosocomial outbreak of RSV infections among inborn infants in the Neonatology clinic of the University Hospital of Obstetrics and Gynaecology “Maichin dom”, Sofia, for the period February–March 2019. The hospital is a tertiary care center for high-risk pregnancies and neonates with 3500–4200 deliveries per year. Healthy babies are cared for in two neonatal wards, with 35 baby beds each. One of the wards uses a rooming-in system. In the other one, the neonates are cared for in the well-baby nursery and stay with their mothers only during breastfeeding. The newborns are admitted to a particular ward, according to the preferences of the parents. Feedback on all problems in the first month after discharge is obtained from the outpatient pediatric practices. The NICU contains 45 beds: 15 intensive and 30 special care beds, separated into 7 boxes. Periods of overcrowding are not excluded. The NICU patients are usually cared for by 8 nurses in each shift. The physicians who work at the NICU are not the same who work at the healthy baby wards. During their daily visits, the parents are encouraged to take part in caring for and feeding the baby if the condition is stable. The practice in our NICU is to stimulate breastfeeding or bottle/ gastric tube feeding with expressed milk using breast-milk fortifiers for the most immature babies.

The epidemic RSV-outbreak, we report here, affected two groups of newborns without contact with each other. Group 1 included 14 healthy term infants, who were cared for in the well-baby nursery, and became symptomatic for LRTI 3 to 5 days after discharge from the hospital. Group 2 included 7 preterm infants with direct (one room), or indirect (caregivers, parents) contact with each other who were treated in the NICU (NICU-patients), and became RSV-positive during the hospital stay. The diagnosis of LRTI was based on standard clinical criteria [17]. Clinical information, including demographic characteristics, symptoms, diagnosis, comorbid illnesses, and the outcome was documented in the hospital case reports. All parents were informed in detail about the disease. Written informed consent for the diagnostics, testing, manipulations, and treatment was obtained. The detection of RSV was performed using Real-Time Polymerase Chain Reaction (PCR). Nasal and pharyngeal specimens were collected and were transported immediately to the National Laboratory “Influenza and Acute Respiratory Diseases”, and were analyzed for viral respiratory pathogens. The results were available the next day. The primers/probes and protocol used in the study were identical to those described by Kodani et al. [18]. Subgroup-specific primers and probes targeting F and N genes of the RSV were applied to determine the RSV-A and RSV-B respectively, using Multiplex Real-Time PCR.

The following measures have been taken to control the epidemic outbreaks:

At the healthy baby ward, strict administration of the standard infection control procedures was carried out: intensification of the disinfection regime; use of gowns, gloves, and masks; regular hand washing and disinfection; restricted visits for relatives other than the mother; denied access for medical staff/parents with respiratory symptoms.

### At the NICU

All infection control procedures mentioned above were applied. As the RSV season was combined with a peak of other seasonal respiratory tract infections, the visits of parents or relatives in the NICU were limited until this outbreak was overcome. Only mothers without respiratory symptoms, who were not discharged from the hospital, were allowed to visit their NICU babies for this period. The RSV-positive infants were separated from the other NICU patients; cohort nursing for these groups was applied. RSV-PCR testing was performed on all 26 patients treated in the special care ward. Regardless of the PCR results immunoprophylaxis with 15 mg/kg palivizumab was administered to all these infants.

## Results

### Group 1

148 term infants were born February 5 to 18, 2019, and treated in the two Neonatal wards for healthy babies. Two to five days after discharge respiratory symptoms were observed in 14 of the babies who were cared for in the baby nursery room. Twelve of these 14 symptomatic babies were re-hospitalized in different Paediatric departments in Sofia with symptoms of LRTI (bronchiolitis). Symptomatic treatment was carried out. The symptoms resolved quickly, no complications were observed and a few days later the infants were discharged home. In 4 of the hospitalized babies, as well as in the 2 outpatient infants, the nasal/throat swabs were tested RSV-PCR positive. RSV etiology was suggested for 8 of the re-hospitalized infants based on epidemiological contact and typical LRTI symptoms, but PCR was not performed. Strict infection control measures were implemented at the healthy baby ward. As a result, the epidemic situation was put under control, and after 18.02.2019 no new cases of RSV-related deterioration in full-term infants were identified. All 148 neonates (Group 1) were closely followed up for respiratory symptoms after their discharge from the 2 neonatal wards. During the reported period, and the entire RSV season there was not a single case of LRTI in infants discharged from the rooming-in system baby ward according to the feedback from the pediatric practices where the babies were followed up.

### The epidemic outbreak in the NICU (group 2)

In the period February 19 to March 15 of 2019 RSV infection was detected in 7 very low and extremely low birthweight in-born infants, aged 1 to 3 months, and treated in the NICU. The clinical data for these patients are summarized in Table 1.

**Table 1:** Data and outcome for RSV-PCR positive preterm infants

Patient (P)	26–27 GW	31–34 GW	BW (g)	Days	BPD	Palivizumab	RSV - PCR I	RSV - PCR II	RSV related symptoms	MV / O2	Bacterial Co-infection	RSV-outcome
**1**	Yes	–	1050	95	Severe	3	(+)	(+)	Severe LRTI Pneumonia	MV	*Enterobacter cloacae*, MRSE (tracheal probe)	Prolonged hospitalization
**2**	Yes	–	840	84	Moderate	1	(+)	(+)	Severe LRTI Pneumonia	MV	Stenotrofomonas maltofilia (tracheal probe)	Prolonged hospitalization
**3**	–	Yes	1410	46	–	–	(+)	No data	LRTI (mild)	O2	–	Recovered
**4**	–	Yes	1410	23	–	–	(+)	(+)	LRTI (moderate) Pneumonia	O2	–	Recovered
**5**	–	Yes	1470	33	–	–	(+)	(+)	LRTI (moderate)	O2	–	Recovered
**6**	Yes	–	1100	69	Moderate	1	–	(+)	LRTI (moderate)	O2	–	Recovered
**7**	Yes	–	850	69	Mild	1	–	(+)	No symptoms	–	–	Healthy

*P* Patient N, *GW* gestational weeks at birth, *BW* birthweight, *Days* days after birth with 1st positive RSV-PCR, *BPD* Bronchopulmonary Dysplasia (O2 at 36 GW), *Palivizumab* number of applications before the 1st positive RSV-PCR test, *RSV-PCR I and II* first and second RSV-PCR test, *MV/O2* Mechanical Ventilation/ Oxygen supplementation, *LRTI* Lower Respiratory Tract Infection;

The first (index) case in the NICU was a 3 months old twin, born at 26 GW, with a birthweight of 1050 g. The other twin was always discharged home, and without respiratory deterioration thereafter. The first NICU patient (P1) had developed severe bronchopulmonary dysplasia (BPD) but was already stable and ready to be discharged home. He had received three injections of palivizumab from the in-hospital palivizumab-immunoprophylaxis course. At the age of 94 days, there was a rapid deterioration with progressive respiratory failure and symptoms of LRTI. Mechanical ventilation was required. The tested nasal and throat swabs were RSV-PCR positive. The X-rays showed infiltrative changes superimposed on a classical BPD image. Since *Staphylococcus epidermidis* and *Enterobacter cloacae* were isolated from tracheal aspirates а complication with ventilator-associated co-infection was discussed and antibiotics were added corresponding to the bacterial sensitivity. Inhaled budesonide and β2-agonists, cardiotonic treatment, diuretics, an additional (booster) palivizumab injection were administered too. After stabilization and extubation, the infant was transferred to the Paediatric department for further treatment of the severe BPD. The second RSV-PCR test performed on day 23 after the disease onset remained positive.

All standard infection control procedures were reinforced aimed to prevent the spread of the RSV infection between the NICU patients. Once RSV was proven in the first patient, we administered palivizumab 15 mg/kg to all 26 preterm babies, who were treated in the special care ward of the NICU and had direct (one room) or indirect (medical staff, parents) contact with the index case. For 22 babies this was the first application. Four infants were injected with a subsequent (booster) dose. One of them was the index case (P1). For three babies the RSV-PCRs were tested positive on the day of the first palivizumab application, and for 3 others – on the following days. However, it is important to emphasize that three of the other extremely preterm NICU patients < 28 GW who had severe BPD and always ongoing palivizumab-prophylaxis remained RSV-negative, and without deterioration during the hospital stay. Four days after the palivizumab injection P2 developed severe respiratory failure with symptoms of bronchiolitis, MV was started. All 26 preterm NICU patients were tested for RSV (33 nasal/throat swabs). Some infants were tested more than once during their hospital stay. We identified 11 positive samples for 7 of the babies, (4 babies were with 2 positive probes). The gestational age of these 7 babies was 26–34 GW, their birthweight was 840-1470 g (Table 1). The babies were put in isolation. In 9 of the samples RSV-B was typified, and in two samples typing was not possible. In 4 babies the second RSV-PCR performed 1 to 3 weeks after the first sample, remained positive, but the clinical symptoms of LRTI were resolved. Due to respiratory deterioration and contact with each other (twins) two babies were re-tested after negative first samples and found to be RSV-PCR positive. In six of all 7 RSV (+) premature infants, symptoms of LRTI (bronchiolitis) occurred, two of them were with severe respiratory failure and needed mechanical ventilation. Three babies were with moderate and one with mild symptoms of LRTI, only one patient (P7) remained asymptomatic. All RSV-positive babies were stabilized, 6 of them – discharged home, one was transferred to the Paediatric department. For the most severely affected infant (P1), the RSV infection significantly deteriorated the clinical course of the BPD and delayed discharge home for more than 2 months.

For Patient 1 the RSV-related symptoms and the RSV-PCR positive test occurred after 3 injections of palivizumab, for P2, P6, and P7 – a few days after the first injection. These infants were at the gestational age of 26–27 GW and with underlying BPD, only one of them (a girl twin) remained without respiratory deterioration. For P3, P4 and P5 palivizumab prophylaxis had not yet been started before the first RSV-positive test. They were at higher gestational age (31–34 GW), without BPD, and presented with mild or moderate clinical symptoms of LRTI.

## Discussion

Here, we describe the first nosocomial RSV-epidemic outbreak in a neonatology clinic since the routine RSV-diagnostics by PCR in Bulgaria was introduced (2013/2014). In the years before this outbreak only isolated cases of RSV infections during the RSV seasons were observed in the NICUs.

RSV is the most common viral pathogen in LRTI infections for infants aged less than 5 years. According to various authors, RSV causes 50–90% of hospitalizations due to bronchiolitis, 5–40% of hospitalizations due to pneumonia, and 10–30% of hospitalizations due to tracheobronchitis [19–21]. In Bulgaria, for the seasons 2015/16, 2016/17, and 2017/18, RSV was identified in 44.5% of the infants with bronchiolitis and 25.1% of those with pneumonia [16]. During the season 2014–2015, serotype A dominated, while over the next three seasons RSV-B was dominant [15, 16]. In our NICU samples, where typing was possible, RSV-B was proven. In Bulgaria, there is a pronounced seasonality of RSV infections during December–March with a peak of diseases in February [15, 16], i.e., the epidemic outbreak described here occurred in the second half of the RSV season.

For the affected full-term infants (Group 1) the symptoms of LRTI appear a few days after discharge from the well-baby nursery. So, they must have acquired the infection during the hospital stay from RSV-positive visitors or healthcare personal with or without respiratory symptoms. Although the mode of transmission of RSV facilitates its spread among the babies [9], the role of the medical staff and gaps in the anti-epidemic regime should be discussed. Unfortunately, in the outbreak, described here, the source of the infection (medical staff or visitors) was not identified. It is important to mention that, there was not a single case of RSV infection among babies cared for in the roaming-in ward. This is likely related to the limited contact with health care personnel, other mothers, or visitors and demonstrates the advantages of the rooming-in system in preventing nosocomial respiratory tract infections in neonatal wards for healthy babies. Furthermore, in such wards, the mothers are actively involved in caring for their infants.

Preterm infants with or without BPD are at increased risk of severe RSV-LRTI during the first 2 years of life. Palivizumab, a monoclonal antibody to the RSV F-protein, was developed for immunoprophylaxis against RSV-disease. Following its license in 1998 palivizumab has been implemented in more and more countries with differing reimbursement criteria, which vary over the years [9, 11, 12, 22, 23]. According to the guidelines of the American Academy of Paediatrics (AAP) until 2014 RSV immunoprophylaxis was administered to preterm infants of gestational age (GA) < 35 GW [22, 24]. Prospective clinical trials have demonstrated palivizumab efficacy of 45–82% against RSV-related hospitalizations [9, 13, 14]. Because of its high price cost-effectiveness studies for different subgroups of high-risk infants were conducted, but they did not provide uniform recommendations for prophylaxis according to gestational age [25, 26]. So, in the following years, in the United States and some EU countries, the reimbursement criteria were limited to the most vulnerable groups: infants born preterm at ≤29 weeks of gestation, infants with BPD, or hemodynamically significant CHD [23, 24, 27]. After the implementation of the RSV-immunoprophylaxis in Bulgaria, 2010, initially covering only the infants with severe BPD, the reimbursement criteria were gradually extended to all infants with GA < 30 GW up to 1 year of age; those < 2 years of age and requiring treatment for BPD within the previous 6 months, or with hemodynamically significant CHD. From season 2019/2020 onwards palivizumab is also reimbursed for preterm infants of gestational age 30 to < 32 GW and < 6 months at the start of the RSV season.

In preterm infants, especially those with BPD, RSV-disease is significantly more severe, with lower respiratory tract involvement or pneumonia. A bacterial infection is often superimposed [21, 27–29]. For our two most critically affected infants, the severe RSV disease was complicated by ventilator-associated infection (P1 and P2).

If the RSV-disease in such high-risk newborns occurs in the NICU, discharge home is delayed. In our population of preterm babies, this period was between 1 week for milder cases and over 2 months for the infant with severe underlying BPD and critical deterioration. To date, there are no uniform recommendations to start palivizumab during the NICU-stay of high-risk infants [10]. So, extremely preterm NICU patients born just before or during the RSV season in Bulgaria (December–March) generally do not receive palivizumab before discharge home. Since the hospital costs would increase significantly the practice in our NICU is to administer off-guideline palivizumab only for extremely preterm infants with the highest risk of severe BPD (need for MV and/or high oxygen requirements during the entire first month of life). Thus, the RSV-prophylaxis for such infants starts about a month after birth with 15 mg/kg of palivizumab monthly. Of our 7 RSV-positive NICU patients, palivizumab has been previously been initiated only for P1, the extremely preterm infant with severe BPD. The other 6 patients were without or low oxygen requirements at 1 month of life. So, they received their first palivizumab injection once the index case was detected.

Among our NICU patients, the RSV infection was most critical in the infant with severe BPD, despite ongoing prophylaxis with palivizumab. The morphological immaturity of the lung and the BPD-related changes might have contributed to the severe course of the disease. Fortunately, despite their low gestational age and co-morbidities, none of the infants in our RSV cohort died. It should be noted that three of the extremely preterm NICU patients with severe BPD had already received one or two injections of palivizumab before the beginning of the RSV outbreak, and they did not get infected. Thus, palivizumab might prevent RSV disease, or help such infants to survive and decrease the number of severe cases.

In the last two decades, several studies on RSV outbreaks in NICUs /Special care baby units with a series of 7–15 preterm infants have been published in the literature [30–34]. Once the first RSV case is identified screening all infants in the NICU, isolating the symptomatic and infected infants, and strict infection control procedures are essential to stop the spread. Dizdar, et al. 2004 reported an RSV outbreak with 15 RSV-positive symptomatic preterm infants, 5 of them (~ 30%) died [30]. During the outbreak reported by Kilani RA (2002) 8 very low gestational age infants were diagnosed with RSV-disease, 4 of them required mechanical ventilation, and one died. At the same time, the 12 term and late preterm infants remained RSV-negative and asymptomatic, probably due to the anti-RSV IgG antibodies that cross the placenta mainly in the last months of pregnancy [35]. In their study, Halasa, et al. summarized the significant negative effects that the RSV outbreak in the NICU had on healthcare delivery, hospital costs, and patient outcome [36]. Analyzing RSV-outbreaks in NICUs most authors conclude that standard infection control measures such as isolation and cohorting, strict hand washing, gowns, and gloves are the main tools to prevent or overcome RSV spread. Palivizumab may help to limit RSV-outbreaks in NICUs [30–32, 34, 37–39], but its use in such situations remains controversial and continues to be discussed. However, the application of palivizumab in the NICU’s praxis as an additional tool to the routine infection control measures is not uncommon [10, 37–39]. Therefore, the need for, and efficacy of palivizumab prophylaxis during RSV outbreaks in NICUs should further be studied.

Another aspect that should be mentioned in the discussion of the reported outbreak is the prolonged shedding of the RSV by the preterm patients: in most of them, the second PCRs tested RSV-positive one to 3 weeks after the first probes, regardless of the clinical recovery. Such a prolonged RSV shedding by high-risk neonates may last for up to 4 weeks [10, 30, 32]. This further complicates the control of the NICU outbreaks and requires isolation of the affected infants until discharge, or until negative PCRs are obtained.

Although full-term infants may also be affected by nosocomial RSV infection, the disease is usually mild. Our full-term symptomatic patients required short-term hospitalization or treatment at home, followed by recovery without complications. In healthy term or near-term (34–37 GW) newborn infants, passive immunity provided by transplacental anti-RSV antibodies would help reduce RSV-related hospitalizations in the first months of life. Active immunization of women during pregnancy, for prevention of severe RSV disease in the neonatal period and early infancy, remains a challenge. The suitable vaccination timing – in the second or third trimester, is also under discussion. It should be sufficient to ensure that the mothers develop immunity, transfer transplacental antibodies, and protect the newborn, including those born preterms [40]. RSV vaccine research and development activities have increased significantly in recent years [21, 41, 42]. Several RSV vaccine clinical trials from Phase I to Phase III with a variety of vaccine strategies, summarized by Rezaee, et al., are currently underway or were recently completed but to date, there is no licensed RSV vaccine for pregnant women [43].

## Conclusions

The reported nosocomial epidemiological outbreak of RSV infections in neonates was the most serious one since the RSV-PCR diagnostic in Bulgaria was introduced. It happened during the second half of the local RSV season. The majority of the affected immature infants had no palivizumab prophylaxis yet before the respiratory symptoms occurred. In extremely preterm infants < 28 GW at high risk of developing BPD, born just before, or at the beginning of the RSV season, it is appropriate to consider reimbursement of early routine in-hospital RSV prophylaxis with palivizumab to prevent RSV disease, or to reduce its severity and complications.

## Data Availability

The main data analyzed in this study are included in this article. All datasets used and/or analyzed during the current study are available from the corresponding author on reasonable request.

## Abbreviations

BPD: Bronchopulmonary Dysplasia
BW: Birth Weight
CHD: Congenital Heart Disease
CLD: Chronic Lung Disease
D: Days
GW: Gestational Weeks at birth
LRTI: Lower Respiratory Tract Infections
MV/O2: Mechanical Ventilation/Oxygen supplementation
NICU: Neonatal Intensive Care Unit
P: Patient
PCR: Polymerase Chain Reaction
RSV: Respiratory Syncytial Virus

## References

[1] Shay DK, Holman RC, Newman RD, Liu LL, Stout JW, Anderson LJ (1999). Bronchiolitis-associated hospitalizations among US children, 1980-1996. JAMA.

[2] Hall CB, Weinberg GA, Iwane MK, Blumkin AK, Edwards KM, Staat MA (2009). The burden of respiratory syncytial virus infection in young children. N Engl J Med.

[3] Shi T, McAllister DA, O'Brien KL, Simoes EAF, Madhi SA, Gessner BD (2017). Global, regional, and national disease burden estimates of acute lower respiratory infections due to respiratory syncytial virus in young children in 2015: a systematic review and modeling study. Lancet.

[4] Lanari M, Prinelli F, Adorni F, Di Santo S, Vandini S, Silvestri M (2015). Study Group of Italian Society of neonatology on risk factors for RSV hospitalization. Risk factors for bronchiolitis hospitalization during the first year of life in a multicenter Italian birth cohort. Ital J Pediatr.

[5] Bennett MV, McLaurin K, Ambrose C, Lee HC (2018). Population-based trends and underlying risk factors for infant respiratory syncytial virus and bronchiolitis hospitalizations. PLoS One.

[6] Hall CB, Weinberg GA, Blumkin AK, Edwards KM, Staat MA, Schultz AF (2013). Respiratory syncytial virus-associated hospitalizations among children less than 24 months of age. Pediatrics..

[7] Blanken MO, Paes B, Anderson EJ, Lanari M, Sheridan-Pereira M, Buchan S (2018). Risk scoring tool to predict respiratory syncytial virus hospitalization in premature infants. Pediatr Pulmonol.

[8] Blanken MO, Rovers MM, Molenaar JM, Winkler-Seinstra PL, Meijer A, Kimpen JL, Dutch RSV Neonatal Network (2013). Respiratory syncytial virus and recurrent wheeze in healthy preterm infants. N Engl J Med.

[9] Resch B (2017). Product review on the monoclonal antibody palivizumab for prevention of respiratory syncytial virus infection. Hum Vaccin Immunother.

[10] Vain NE (2020). Nosocomial respiratory viral infection in the neonatal intensive care unit. Am J Perinatol.

[11] Palivizumab, a humanized respiratory syncytial virus monoclonal antibody, reduces hospitalization from respiratory syncytial virus infection in high-risk infants. The IMpact-RSV Study Group. Pediatrics. 1998;102(3 Pt 1):531–7. 9724660

[12] Torchin H, Rousseau J, Marchand-Martin L, Truffert P, Jarreau PH, Ancel PY (2018). Palivizumab administration in preterm infants in France: EPIPAGE-2 cohort study. Arch Pediatr.

[13] Feltes TF, Cabalka AK, Meissner HC, Piazza FM, Carlin DA, Top FH (2003). Cardiac Synagis study group. Palivizumab prophylaxis reduces hospitalization due to respiratory syncytial virus in young children with hemodynamically significant congenital heart disease. J Pediatr.

[14] Anderson EJ, Carosone-Link P, Yogev R, Yi J, Simões EAF (2017). Effectiveness of Palivizumab in high-risk infants and children: a propensity score weighted regression analysis. Pediatr Infect Dis J.

[15] Korsun N, Angelova S, Tzotcheva I, Georgieva I, Lazova S, Parina S (2017). Prevalence and genetic characterization of respiratory syncytial viruses circulating in Bulgaria during the 2014/15 and 2015/16 winter seasons. Pathog Glob Health.

[16] Korsun N, Angelova S, Trifonova I, Georgieva I, Voleva S, Tzotcheva I (2019). Viral pathogens associated with acute lower respiratory tract infections in children younger than 5 years of age in Bulgaria. Braz J Microbiol.

[17] Carande EJ, Pollard AJ, Drysdale SB. Management of Respiratory Syncytial Virus Bronchiolitis: 2015 survey of members of the European Society for Paediatric Infectious Diseases. Can J Infect Dis Med Microbiol 2016;2016:9139537. 10.1155/2016/9139537. 10.1155/2016/9139537 PMC5093249 27840650

[18] Kodani M, Yang G, Conklin LM, Travis TC, Whitney CG, Anderson LJ (2011). Application of TaqMan low-density arrays for simultaneous detection of multiple respiratory pathogens. J Clin Microbiol.

[19] Calvo C, Pozo F, García-García ML, Sanchez M, Lopez-Valero M, Pérez-Breña P (2010). Detection of new respiratory viruses in hospitalized infants with bronchiolitis: a three-year prospective study. Acta Paediatr.

[20] Esposito S, Daleno C, Prunotto G, Scala A, Tagliabue C, Borzani I (2013). Impact of viral infections in children with community-acquired pneumonia: results of a study of 17 respiratory viruses. Influenza Other Respir Viruses.

[21] Mazur NI, Martinón-Torres F, Baraldi E, Fauroux B, Greenough A, Heikkinen T (2015). Respiratory syncytial virus network (ReSViNET). Lower respiratory tract infection caused by respiratory syncytial virus: current management and new therapeutics. Lancet Respir Med.

[22] Committee on Infectious Diseases (2009). From the American Academy of Pediatrics: policy statements--modified recommendations for use of palivizumab for prevention of respiratory syncytial virus infections. Pediatrics.

[23] American Academy of Pediatrics Committee on Infectious Diseases; American Academy of Pediatrics Bronchiolitis Guidelines Committee (2014). Updated guidance for palivizumab prophylaxis among infants and young children at increased risk of hospitalization for respiratory syncytial virus infection [published correction appears in Pediatrics. 2014;134(6):1221]. Pediatrics.

[24] Belleudi V, Trotta F, Pinnarelli L, Davoli M, Addis A (2018). Neonatal outcomes following new reimbursement limitations on palivizumab in Italy. Arch Dis Child.

[25] Meissner HC, Kimberlin DW (2013). RSV immunoprophylaxis: does the benefit justify the cost?. Pediatrics.

[26] Ginsberg GM, Somekh E, Schlesinger Y (2018). Should we use Palivizumab immunoprophylaxis for infants against respiratory syncytial virus? - a cost-utility analysis. Isr J Health Policy Res.

[27] Drysdale SB, Green CA, Sande CJ (2016). Best practice in the prevention and management of pediatric respiratory syncytial virus infection. Ther Adv Infect Dis.

[28] De Brasi D, Pannuti F, Antonelli F, de Seta F, Siani P, de Seta L (2010). Therapeutic approach to bronchiolitis: why pediatricians continue to overprescribe drugs?. Ital J Pediatr.

[29] Baraldi E, Lanari M, Manzoni P, Rossi GA, Vandini S, Rimini A (2014). Inter-society consensus document on treatment and prevention of bronchiolitis in newborns and infants. Ital J Pediatr.

[30] Dizdar EA, Aydemir C, Erdeve O, Sari FN, Oguz S, Uras N (2010). Respiratory syncytial virus outbreak defined by rapid screening in a neonatal intensive care unit. J Hosp Infect.

[31] Cox RA, Rao P, Brandon-Cox C (2001). The use of palivizumab monoclonal antibody to control an outbreak of respiratory syncytial virus infection in a special care baby unit. J Hosp Infect.

[32] Abadesso C, Almeida HI, Virella D, Carreiro MH, Machado MC (2004). Use of palivizumab to control an outbreak of syncytial respiratory virus in a neonatal intensive care unit. J Hosp Infect.

[33] Visser A, Delport S, Venter M (2008). Molecular epidemiological analysis of a nosocomial outbreak of respiratory syncytial virus associated pneumonia in a kangaroo mother care unit in South Africa. J Med Virol.

[34] Saadah LM, Chedid FD, Sohail MR, Nazzal YM, Al Kaabi MR, Rahmani AY (2014). Palivizumab prophylaxis during nosocomial outbreaks of respiratory syncytial virus in a neonatal intensive care unit: predicting effectiveness with an artificial neural network model. Pharmacotherapy.

[35] Kilani RA (2002). Respiratory syncytial virus (RSV) outbreak in the NICU: description of eight cases. J Trop Pediatr.

[36] Halasa NB, Williams JV, Wilson GJ, Walsh WF, Schaffner W, Wright PF (2005). Medical and economic impact of a respiratory syncytial virus outbreak in a neonatal intensive care unit. Pediatr Infect Dis J.

[37] Kurz H, Herbich K, Janata O, Sterniste W, Bauer K (2008). Experience with the use of palivizumab together with infection control measures to prevent respiratory syncytial virus outbreaks in neonatal intensive care units. J Hosp Infect.

[38] O'Connell K, Boo TW, Keady D, Niriain U, O'Donovan D, Commane M (2011). Use of palivizumab and infection control measures to control an outbreak of respiratory syncytial virus in a neonatal intensive care unit confirmed by real-time polymerase chain reaction. J Hosp Infect.

[39] Hammoud MS, Al-Taiar A, Raina A, Elsori D, Al-Qabandi S, Al-Essa M (2016). Use of palivizumab with other infection control measures to control respiratory syncytial virus outbreaks in neonatal care units. J Trop Pediatr.

[40] Saso A, Kampmann B (2016). Vaccination against respiratory syncytial virus in pregnancy: a suitable tool to combat global infant morbidity and mortality?. Lancet Infect Dis.

[41] Giersing BK, Karron RA, Vekemans J, Kaslow DC, Moorthy VS (2019). Meeting report: WHO consultation on respiratory syncytial virus (RSV) vaccine development, Geneva, 25-26 April 2016. Vaccine.

[42] Vekemans J, Moorthy V, Giersing B, Friede M, Hombach J, Arora N (2019). Respiratory syncytial virus vaccine research and development: World Health Organization technological roadmap and preferred product characteristics. Vaccine.

[43] Rezaee F, Linfield DT, Harford TJ, Piedimonte G (2017). Ongoing developments in RSV prophylaxis: a clinician's analysis. Curr Opin Virol.

